# Reference Values of Physical Performance in Handball Players Aged 13–19 Years: Taking into Account Their Biological Maturity

**DOI:** 10.3390/clinpract14010024

**Published:** 2024-02-06

**Authors:** Chirine Aouichaoui, Samir Krichen, Mohamed Tounsi, Achraf Ammar, Oussama Tabka, Salem Chatti, Monia Zaouali, Mohamed Zouch, Yassine Trabelsi

**Affiliations:** 1 Research Laboratory, Exercise Physiology and Physiopathology: From Integrated to Molecular “Biology, Medicine and Health”, LR19ES09, Faculty of Medicine of Sousse, Sousse University, Sousse 4000, Tunisia; chirineaouichaoui@yahoo.com (C.A.); samirkrichen2@yahoo.fr (S.K.); m.tounsi@hotmail.fr (M.T.); oussamatabka@hotmail.fr (O.T.); chattisalem23@gmail.com (S.C.); zaoualimonia@yahoo.fr (M.Z.); mohamedzouch@yahoo.fr (M.Z.); trabelsiyassine@yahoo.fr (Y.T.); 2 High Institute of Sport and Physical Education of Ksar Saïd, University of Manouba, Mannouba 2010, Tunisia; 3 Department of Training and Movement Science, Institute of Sport Science, Johannes Gutenberg-University Mainz, 55122 Mainz, Germany; 4 High Institute of Sport and Physical Education of Sfax, University of Sfax, Sfax 3029, Tunisia; 5 Research Laboratory, Molecular Bases of Human Pathology, LR19ES13, Faculty of Medicine of Sfax, University of Sfax, Sfax 3000, Tunisia

**Keywords:** physical parameters, anthropometry, percentile data, peak height velocity, handball players

## Abstract

Biological maturity status significantly influences success in handball, impacting an athlete’s performance and overall development. This study aimed to examine the anthropometric and physical performance variables concerning age and maturity status, establishing reference values for physical performance among Tunisian players. A total of 560 handball players (309 males and 251 females aged 13–19 years) were categorized based on maturity status: early (*n* = 98), average (*n* = 262), and late (*n* = 200), determined through Mirwald and colleagues’ equations. Anthropometric, physical fitness, and physiological data were collected for reference value creation. Our findings revealed significantly higher anthropometric parameters (*p* = 0.003) in late-maturing athletes compared to their early-maturing counterparts. Post-pubertal athletes showed significantly superior (*p* = 0.002) jumping ability, change of direction, and aerobic performance compared to their pre-pubertal peers. Additionally, male athletes outperformed females in both fitness (*p* = 0.001) and aerobic (*p* = 0.001) performance. A notable age-by-maturity interaction emerged for most performance outcomes (η^2^ ranging from 0.011 to 0.084), highlighting increased sex-specific differences as athletes progressed in age. Percentile values are provided for males and females, offering valuable insights for coaches and sports scientists to design personalized training programs. Understanding a player’s performance relative to these percentiles allows trainers to tailor workouts, addressing specific strengths and weaknesses for enhanced development and competitiveness.

## 1. Introduction

Team handball is an Olympic sport ball game influenced by the performance of each athlete, as well as tactical aspects and team interaction [1]. For decades, it has been the second most widely followed sport globally, practiced on all continents. It has its own World Cup and is an official competition in the games [1]. In addition, handball is an intense sport that involves physical contact between athletes, requiring high somatic and technical capabilities [2,3]. Anthropometric parameters, speed, agility, strength, muscle power, and technical skills are all considered crucial factors for successful participation in handball leagues at all levels [4]. Notably, the strength and power of both the lower and upper limb muscles play a significant role in throwing, sprinting, and jumping for handball players [5]. In this game, movement patterns are characterized by intermittent and continuous changes in response to fast-paced offensive and defensive actions [6,7].

Handball is a sport with high anaerobic demands, as indicated by a study conducted in adults [8]. However, the sport’s performance is largely determined by players’ aerobic capacity with repetitive high-intensity movements supplied by anaerobic metabolism [9]. Players typically cover a distance of approximately four to six kilometers at an intensity near 80% to 90% of their maximum heart rate [9]. Several researchers have examined factors associated with performance in handball players, including gender, anthropometric variables, playing position, and experience [10,11]. Notably, performance in handball is influenced by factors such as sexual, skeletal, and somatic maturation, which are crucial components for evaluating growth spurt. The duration and timing of this growth spurt can vary significantly among individuals [12]. Furthermore, specific anthropometric measurements, along with certain physical performance metrics, prove to be valuable for talent identification [12]. In youth handball teams, players who have attained advanced biological maturation are generally heavier and taller, displaying superior performance in tests that assess strength, speed, and power [2]. Conversely, several studies [13,14] have indicated an inverse relationship between physical activity and the timing of sexual maturation. Physical activity tends to decrease as both chronological and biological age increases, regardless of gender [13,14]. This means that as individuals grow older and go through the process of biological maturation, their level of physical activity tends to decrease [13,14].

Peak height velocity (PHV) is a period of peak stature development during a growth spurt. It is, rather than chronological age, used to describe changes in body composition. Therefore, a comprehensive assessment of growth requires determining the timing of biological maturation. Puberty not only illustrates the transition to maturity but also includes many physiological and bodily transformations. Biological maturation and pubertal growth are dynamic processes influenced by various environmental, nutritional, and genetic factors [15]. Moreover, in several studies and across various sports, inter-individual differences in biological maturation are rarely considered. Hammami et al. [16] demonstrated that there are differences in the performance of European adolescent males in tests of running speed and change-of-direction speed in pre-PHV and post-PHV male players. In contrast, Di Giminiani and Visca [17] demonstrated that while soccer training led to improvements in the physical performance of young Italian players, they did not find any associations between changes in physical performance rates and biological maturation.

Maturation can influence changes in jump performance among young male swimmers between the pre-peak and post-peak height velocity stages [18]. Indeed, adaptations were less pronounced during the pre-peak height velocity stage compared to the post-peak height velocity stage [18].

The relative age effect among handball players was detected across different playing positions and various physical performance parameters [1,19,20]. In fact, Hammami et al. [21] revealed a significant chronological age effect in Tunisian male handball players. Older players displayed greater body dimensions and considerably superior results on diverse physical assessments compared to their younger counterparts [21]. However, there is limited knowledge regarding the impact of biological maturation on the anthropometric characteristics and physical parameters of Tunisian handball players, which are crucial factors for improving handball performance [22]. Furthermore, the establishment of reference values for physical parameters based on maturity status in adolescent handball players is of great importance. This allows coaches to control various training programs, optimize the effectiveness of different training regimens, and enhance overall performance [10]. Having percentile values for muscular strength parameters may assist in facilitating health enhancement and estimating the percentage of adolescents with high or low levels of muscular strength [10]. Identifying each athlete’s PHV is essential for evaluating individual performance. The assessment of the state of biological maturity should be considered during talent identification. A predefined level of anaerobic and aerobic potential should be one of the criteria when screening candidates for competitive handball.

To our knowledge, no studies have evaluated these variables in male and female Tunisian players, taking into account their maturation status. Understanding how biological maturity status in Tunisian adolescent handball players affects their anthropometric parameters and physical performance is important, along with establishing percentile values according to gender and maturity status. Percentile values are used to assess physical fitness, providing a standardized method for comparing individuals or groups. Athletes can utilize these benchmarks to gauge their standing within a specific demographic, assisting trainers in tailoring training programs and setting realistic goals. Therefore, this study aims to (1) analyze the evolution of anthropometric parameters and physical performance by chronological age and maturity status in Tunisian handball players aged 13 to 19 and (2) establish specific percentile values for physical performance based on maturity status and gender in Tunisian handball players.

Regardless of gender, we hypothesize that late-maturing players will demonstrate significantly higher values for both anthropometric and performance parameters compared to on-time and early-maturing players across consecutive maturity groups.

## 2. Materials and Methods

### 2.1. Players

A total of 560 Tunisian handball players (309 males and 251 females) were included in the study. We used the formula for sample size calculation to calculate the necessary sample size. In this study, with a 5% α error level, Z_α_ was set at 1.96, and Z_1−β_ (representing the study’s power) was 0.8416. The estimated statistical variance (σ^2^ = 1) and standard deviation (SD = 1) represent the variability in our population and were based on previous studies [6,16,21]. Delta (Δ) corresponds to the minimum meaningful effect size we aimed to detect in our study. Based on the same studies, Δ was estimated to be equal to 0.43 for our research [6,16,21]. We rounded the population number to the nearest whole number. Therefore, for this research, we required a minimum of 30 subjects in each group (*n*) to achieve an 80% power level for detecting a difference of 0.43 [23].

Players aged between 13 and 19 years were randomly selected from different handball teams. These players participated in the national junior and senior championship leagues each week. Before the study, both players and their families were informed of the research objectives, the protocol, and the investigation procedures. They were asked to provide us with written consent, which we obtained after the parents or players had signed it. None of the subjects were taking medications that could influence the results of the current study. All players were examined by the medical team and were cleared for participation in the team activities.

Prior to conducting the investigation, the research protocol was validated by the ethics committee of Farhat Hached Hospital in Sousse (IRB provided by OHRP: IRB00008932). The research was conducted in compliance with the ethical standards outlined in the Helsinki Declaration of the World Medical Association, which was initially established in 1964 and modified in 2013 [24]. It is important to note that 70 participants withdrew before the research began for individual reasons (5 from each category), and their statistics were not mentioned in the statistical study.

### 2.2. Anthropometric Measurements

The sitting and standing heights were assessed using a stadiometer (Harpenden Portable Stadiometer, UK) with precision to the nearest 0.1 cm. The players were weighed in minimal clothing on a digital scale (Harpenden Balance Scale, UK) accurate to the nearest 0.1 kg. The leg length was determined by calculating the difference between the standing and sitting height. The body mass index (BMI) was calculated as the ratio of weight (kg) to the square of height (meters). The lower body impedance was measured using a Tanita TBF-604 body fat monitor/scale (Tokyo, Japan). This device requires input data such as the subject’s body mass, standing height, and gender. The subject then stood on the scale, which incorporated detector electrodes and sources on the plantar surfaces of both feet to measure the lower body impedance and calculate the percentage of body fat [25].

The absolute body fat weight was determined as follows: fat weight (kg) = fat percentage × (weight/100). The fat-free weight (kg) was calculated by subtracting the fat weight from the total body weight.

The wingspan was measured using a tape measure, with the measurement taken from fingertip to fingertip when the arms were held parallel to the ground. Players stood up straight with their backs against a wall and their arms stretched out to their sides, perpendicular to their bodies. Handspan is an assessment of hand size involving the measurement of the width of the hand when the fingers are spread out. The procedure entails placing the hand’s palm down on a flat surface, and the fingers are extended as far as possible to gauge the linear distance between the outer edge of the thumb and the outer edge of the little finger [25]. All anthropometric measurements were conducted in the afternoon towards the end of the week, facilitated by a specialized physician.

### 2.3. Procedure

**Figure 1:**
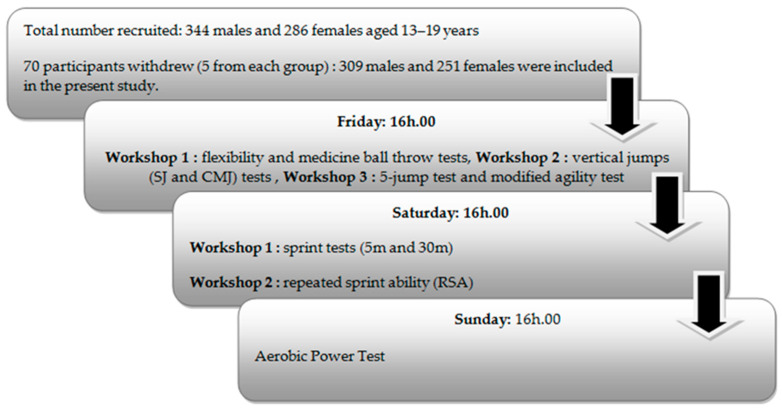
Study design for adolescent Tunisian handball players aged 13–19 years.

During the competitive season of the previous year, specifically in the month of February, the study was conducted over a period of three days (see Figure 1). Prior to the commencement of the experimentation, players refrained from exercise on the day before testing and abstained from consuming caffeine beverages for at least 4 h prior to testing. The players were allowed to drink water up to 2 h before the test.

In terms of meals, the players were instructed to avoid eating heavy meals for at least 3 h before the testing sessions. Prior to each test, the players conducted a standard warm-up lasting 15 to 20 min. All analyses were conducted in a single session, commencing at 4:00 p.m. The selection of this timing was informed by research indicating optimal performance during the late afternoon for anaerobic tests, aligning with the hours of training sessions, as determined by Chtourou et al. [26], with the exception of the aerobic test, which was conducted in a separate session.

Day 1: Participants were asked to measure their flexibility, complete a medicine ball throw, and realize physical tests, which included the squat jump (SJ), countermovement jump (CMJ), 5-jump test, and a modified agility test in the indoor handball court.

Day 2: The second day of the intervention focused on conducting the repeated sprint ability (RSA) test and sprint tests in the indoor handball court. 

Day 3: The third day of the intervention was dedicated to conducting the aerobic power test to assess the maximum aerobic speed in the indoor handball court. 

Four investigators ensured the completion of all the tests. The participants received verbal encouragement, and all measurements were collected and performed under the same conditions. The errors of measurement were <1 mm. The coefficient of variation (CV) for test-retest reliability was 4.2%, with test-retest 95% intra-class correlation coefficient (ICC) values for the testing procedure ranging from 0.98 to 0.99 [27].

#### 2.3.1. Flexibility

The flexibility test was performed using a digital anteflexion meter (TKK-5403) to assess the flexibility of the lower back and hamstrings [28]. The TKK-5403 FLEXION-D is a digital device designed to measure the extent of forward body bending. It can display each measured value and the larger value of two measurements. The measuring range was from −20.0 cm to +35.0 cm. The players executed the test without footwear, positioning themselves with their legs extended and feet close together while standing on a platform. The participants were instructed to bend over, utilizing their full range of motion, and to maintain the position with extended knees, fingers, and arms for a minimum of two seconds during the test [28].

#### 2.3.2. Medicine Ball Throw Tests

Throwing is a fundamental skill in handball. Strength and power in the upper extremities were assessed through medicine ball throw tests. The procedure involves bringing a 3 kg medicine ball behind the head and then propelling it forward as far as possible. The players were instructed to hold the medicine ball in both hands in front of their chests with their elbows at the same level as their hands. Their task was to push the ball as far as they could, and the distance was measured using a measuring tape [29].

The players were permitted and encouraged to step forward over the line after releasing the ball to maximize the throw’s distance and velocity. Each player had three attempts, and the throw with the highest average velocity was chosen for analysis [29].

#### 2.3.3. Jump Assessments

Jumping performance was evaluated using the Optojump Next device (Microgate SRL, Italy), connected to a computer for data recording, including jump height, power, and contact and flight times. Vertical jumps were measured using both squat jump (SJ) and countermovement jump (CMJ) protocols. Before the specific jump test, handball players were allowed two practice jumps. The best of three attempts measured to the nearest centimeter was recorded, with a two-minute rest period between jumps to ensure adequate recovery. Throughout all the jumps, players maintained their hands on their hips to eliminate the influence of arm swing impulse [30].

In the squat jump (SJ) test, the players were instructed to descend and hold a knee position (approximately a 90° knee angle) for three seconds. Following the count of three, the player was directed to jump as high as possible without any countermovement before the execution of the jump. A successful trial was one where there was no sinking or countermovement before the jump [30].

For the countermovement jump (CMJ) assessment, the players began in a standing position and, before jumping, executed a countermovement until the knee was flexed to approximately 90°. They were then instructed to descend as rapidly as possible and jump as high as possible during the subsequent concentric phase. Consistent verbal encouragements were provided to maintain high motivation in these groups [30]. Each participant performed three consecutive experimental trials for each jump, and the best values for each jump were retained for further analysis. The players performed jumps in running shoes and comfortable clothing.

#### 2.3.4. Five-Jump Test (5JT)

The five-jump test contains five consecutive strides with both feet joined at the initiation and end of the jumps. The horizontal jumping test is commonly quantified in absolute terms, representing the total distance covered in meters [31].

#### 2.3.5. Modified Agility *t*-Test

The modified agility *t*-test is designed to assess agility, involving various running techniques like forward, backward, and lateral movements. Times for agility tests were measured using Photocells Witty (Microgate). This test specifically evaluates directional speed changes, including activities such as forward sprints, left and right shuffling, and backpedaling. The modified agility *t*-test adhered to the same protocol as the *t*-test, with alterations to the covered distance and measurements of the inter-cone distance. Nevertheless, the number of directional changes remained consistent. The players were encouraged to execute directional changes as swiftly as possible, resulting in an approximate 45° change in direction. The recorded score for this test was determined by the best performance in the last two trials during the test-retest session [32].

#### 2.3.6. Sprint Tests

Sprint tests were conducted to evaluate speed. Before these tests, participants underwent warm-ups, which included a run of low intensity followed by stretching exercises controlled by coaches. The dynamic exercises were specifically designed to enhance the flexibility of the muscle groups essential for sprints, including the flexors and extensors of the knee, hip, and ankle joints [33].

During the 5-m and 30-m sprint tests, the players were measured using photocell gates, specifically the Witty system, which is a portable timing system by Microgate [34,35]. When ready to sprint, the subjects initiated a sprint from a standing start positioned 0.5 m behind the starting line. The starting stance was consistent for each subject. The timing began as the subject crossed the first gate at the 0 m mark, with split times recorded at 5 m and 30 m. Each participant underwent three trials, separated by at least 5 min of rest, and the fastest time, measured to the nearest 0.01 s, was used as the speed score. The athletes performed the sprints in tight-fitting clothing and spiked track shoes. The participants were provided with verbal encouragement to exert maximum effort during sprints [33]

#### 2.3.7. Repeated Sprint Ability (RSA)

The repeated sprint ability (RSA) test involved six repetitions of maximal 6 × 15 m shuttle sprints, with 25 s of standing recovery in between. Three seconds prior to the start of each sprint, the subjects assumed the starting position and awaited the beginning signal. Verbal encouragement was consistently provided to each subject during the tests. Each sprint shuttle involved a single change in direction and was timed using a Photocells Witty (Microgate) system placed at a height of 1 m. After crossing the finishing line, players were encouraged to decelerate as soon as possible, walk back slowly, and wait for the next sprint [36]. 

#### 2.3.8. Twenty-Meter Shuttle Run Test

The maximal multistage shuttle run test, initially described by Léger and Lambert [37] and later modified by Léger et al. [38], was employed to estimate the maximal oxygen consumption based on the maximal aerobic speed. The players were directed to run between two lines spaced 20 m apart, receiving encouragement to sustain running for as long as possible. The pace was regulated by a cassette tape emitting tones at determined intervals. The initial speed was set at 8.5 km.h^−1^ for the first minute and increased by 0.5 km.h^−1^ after each one-minute stage. The test concluded when the athlete could no longer maintain the required pace due to muscle fatigue. At the test’s conclusion, the number of fully completed shuttles was recorded for data analysis [29]. The sores from the last stage were then converted to predict the maximal oxygen uptake (VO_2max_), expressed as ml of oxygen consumed per kilogram of body weight and per minute (mL.kg^−1^.min^−1^). The maximal aerobic speed (MAS) is the lowest speed that enables the attainment of VO_2max_, representing the maximum aerobic power level for a player [38].

### 2.4. Maturity Status

Peak height velocity (PHV) is simply the period during which an adolescent experiences the fastest upward growth in stature. For the maturity offset assessment, representing maturational time-points based on the distance (in years) from the age at which a child will achieve their PHV, we employed a non-invasive method, specifically, the equations developed by Mirwald et al. [39]. These equations use morphological measures, such as leg length, standing height, sitting height, weight, and chronological age, along with specific coefficients for each gender, to calculate the maturity offset [39]. They were as follows:

For males: maturity offset = −9.236 + (0.0002708 × leg length × sitting height) − (0.001663 × age × leg length) + (0.007216 × age × sitting height) + (0.02292 × weight by height ratio).

For females: maturity offset = −9.376 + (0.0001882 × leg length × sitting height) + (0.0022 × age × leg length) + (0.005841×age × sitting height) − (0.002658 × age × weight) + (0.07693 × weight by height ratio)

After calculating the maturity offset, we categorized maturity into three groups based on the value of maturity status for each player [40]:

(1) Pre-PHV/early-maturing (−3 years to >−1 year from PHV);
(2) Circa PHV/on-maturing (−1 to +1 year from PHV);
(3) Post-PHV/late-maturing (>1 to +3 years from PHV).

For example, if the maturity offset was −2, it indicated that the individual was two years before age at PHV. Consequently, we classified this player as pre-PHV.

### 2.5. Statistical Analysis

Descriptive statistics for all parameters were shown as the mean ± standard deviation (SD). The normality of the values was assessed using the Kolmogorov-Smirnov test. Within each test cluster, differences in anthropometric and performance measures based on age category and/or maturity status were analyzed through a multivariate analysis of variance (MANOVA). To account for multiple comparisons, all MANOVA analyses were adjusted using the Bonferroni correction. The effect sizes for the MANOVA test results were quantified in terms of eta squared values (η^2^) and categorized as follows: <0.06 denoting a low effect, 0.06 to 0.14 indicating a moderate effect, and >0.15 representing a large effect [41]. Percentile analyses were conducted separately for males and females based on their maturity classification. The 5th, 10th, 25th, 50th, 75th, 90th, and 95th percentiles were calculated. For each test-retest measured parameter, 95% confidence intervals (95% CI) were determined. All analyses were performed using SPSS 18.0 software, with the significance level set to *p* < 0.05.

## 3. Results

### 3.1. Anthropometric Parameters and Physical Performance Differences According to Age and Sex

**Figure 2:**
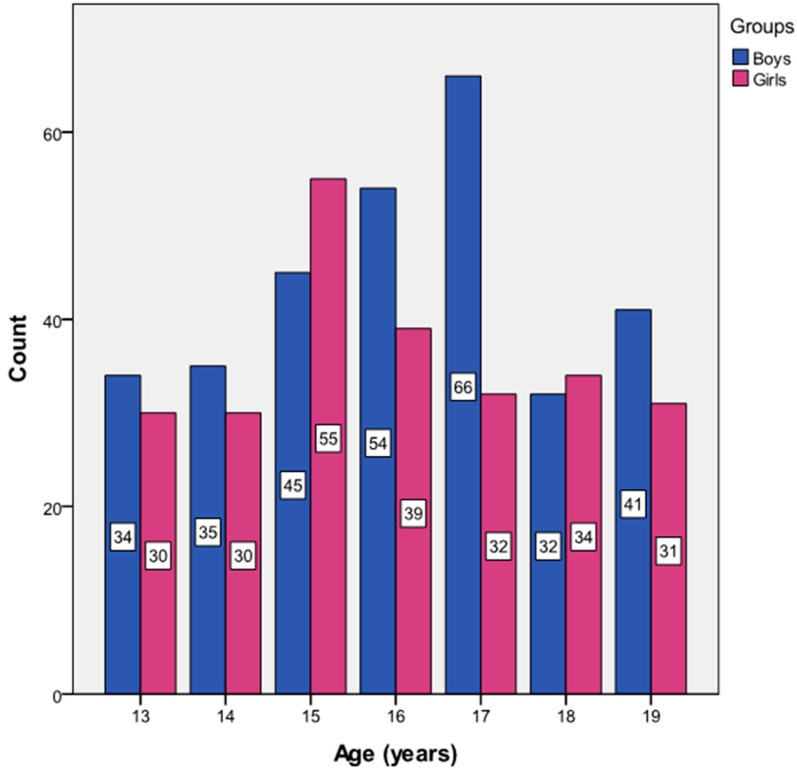
Distribution in Tunisian handball players according to chronological age and gender.

The distribution of count data in both genders according to chronological age is shown in Figure 2.

Descriptive statistics of the physical characteristics and body compositions of Tunisian handball players are summarized in Table 1. Descriptive statistics for the physical and physiological performance of Tunisian handball players are presented in Table 2. Independent of players‘ gender, an increase in the anthropometric and physiological parameters was observed as chronological age increased, with the exception of speed and RSA times, which decreased with age (as shown in Table 1 and Table 2). Significantly, there were notable differences in the majority of parameters between both sexes (*p* < 0.05) from the age of 14. More precisely, the male athletes were significantly taller and heavier in comparison to the female athletes (see Table 1). Furthermore, the boys outperformed the girls in the performance evaluation tests (as shown in Table 2). The jump heights of the handball players during the countermovement jump (CMJ) test were significantly higher (*p* < 0.05) than during the SJ test (as indicated in Table 2). 

### 3.2. Anthropometric Characteristics and Physical Performance According to Maturity Status and Sex

**Figure 3:**
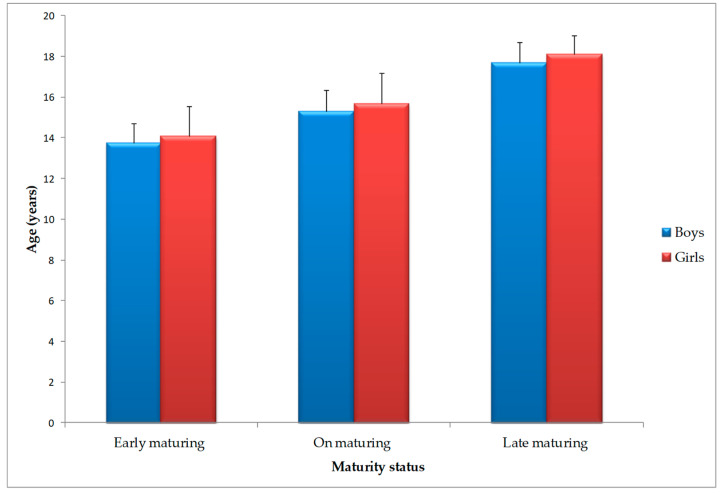
Distribution of Tunisian handball players according to maturity status and gender.

The distribution of “early”, “on”, and “late” maturing handball players, separated by gender, is displayed in Figure 3.

Descriptive statistics for the anthropometric characteristics and physiological performance among Tunisian handball players, grouped by maturity status and gender, are provided in Table 3 and Table 4.

Based on the classification of maturity status, which is determined by years from peak height velocity (PHV), 46.78% of both male and female players were categorized as “on-maturing”, 17.50% were characterized as “early-maturing”, and 35.71% were characterized as “late-maturing”.

In terms of anthropometry, all players were classified as “early”, “on”, or “late” maturing. Significant differences between maturity categories were observed for body mass, standing height, sitting height, leg length, body mass index, fat mass, fat-free mass, boys’ body fat percentage, wingspan, and boys’ span. For example, the post-hoc analysis indicated that body height and mass were significantly higher in late-maturing players compared to early-maturing players. However, no significant differences between maturity categories (*p* > 0.05) were observed for girls’ body fat percentage and girls’ span (refer to Table 3)

In terms of physical performance, all players were categorized as “pre-pubertal”, “pubertal”, or “post-pubertal”. Our analysis revealed significant differences among the three maturity categories for all performance parameters (*p* < 0.05), with the exception of maximum oxygen uptake (VO_2max_), SJ height, and CMJ height for girls (see Table 4). The post-hoc analysis demonstrated that later-maturing players showed significantly higher maximum aerobic velocity, SJ height, CMJ height, and five-jump test results compared to their earlier maturing players. Similarly, faster sprint times over 5 m and 30 m and better agility and RSA tests were observed for the later maturing group compared to the earlier-maturating players.

A significant gender effect was observed for all anthropometric and performance variables in the late-maturing players. Independent of players’ gender, an increase in performance was noted across consecutive maturity groups (pre-pubertal < pubertal < post-pubertal). 

### 3.3. Difference in Anthropometric Variables According to Age Category and/or Maturity Status per Test Cluster

A MANOVA was used to analyze the differences in the anthropometric and performance variables according to age category and/or maturity status within each test cluster. The results of the main and interaction effects, as well as effect sizes (η^2^) are displayed in Table 5 and Table 6. Generally, the calculated effect sizes (η^2^) are less than 0.06, indicating small effects, between 0.06 and 0.14 as moderate effects, and greater than 0.15 as large effects for both anthropometric and physical parameters.

#### 3.3.1. Interaction Effects of Maturity and Chronological Age

Our analyses revealed significant and meaningful age-by-maturity interactions in nearly all anthropometric parameters (standing height, sitting height, fat-free mass, body fat percentage, and span) (as shown in Table 5). Additionally, significant age-by-maturity interactions were observed for SJ height, the 5-jump test, the agility test, and the medicine ball throw (*p* < 0.05) (refer to Table 6).

#### 3.3.2. Effects of Chronological Age

Significant main effects of chronological age were observed for all anthropometric variables (*p* < 0.01; η^2^ ranging from 0.02 to 0.1), except for body mass, standing height, and fat-free mass (as indicated in Table 5). Furthermore, we identified a main age effect (*p* < 0.01; η^2^ ranging from 0.02 to 0.1) for the physical parameters, except for the maximum oxygen uptake (VO_2max_), SJ height, CMJ height, and medicine ball throw. 

#### 3.3.3. Effects of Maturity Status

In relation to the maturity status of handball players, statistically significant main effects were observed for all anthropometric and physiological parameters (*p* < 0.01; η^2^ ranging from 0.02 to 0.4), with the exception of the leg length and flexibility (*p* > 0.05; η^2^ = 0.006 and 0.002, respectively).

### 3.4. Percentile Values According to Maturity Status and Sex 

The 5th, 10th, 25th, 50th, 75th, 90th, and 95th percentile values, specific to gender and categorized by maturity status, for the jump tests (CMJ, SJ, and 5-jump tests), change-of-direction speed tests, medicine ball throw, and flexibility of Tunisian handball players aged 13 to 19 are presented in Table 7.

## 4. Discussion

The main findings of the present study were as follows: (1) the anthropometric and physical performance parameters significantly improved with chronological age, and (2) the maturity status and gender had a significant impact on the anthropometric parameters and physical performance, with late-maturing male players demonstrating significantly higher values than their female counterparts. These sex-specific differences increased with advancing age and maturity categories. This study provides reference values for physical performance based on maturity status and gender in Tunisian handball players aged 13–19 years.

### 4.1. Chronological Age and Gender-Specific Anthropometric and Physical Parameters

The present study revealed that all of the anthropometric variables significantly increased with chronological age. These findings are in accordance with the study performed by Tounsi et al. [42], which reported similar results in healthy Tunisian adolescents aged 13–19 for both sexes. 

The pathway from adolescence into adulthood inevitably leads to body growth as well as cognitive and somatic development. The growth rate peaks at approximately 14 years in boys and 12 years in girls and gradually decreases, eventually ceasing with the attainment of adult stature. Consequently, physical development depends on and is influenced by maturation and growth, thus affecting physical and physiological parameters [16,43,44].

During adolescence, boys show improvements in performance that extend into early adulthood, whereas girls’ strengths tend to plateau around the time of puberty and then tend to decrease thereafter. This difference could be explained by an increase in girls’ percentage of body fat. This increase in body fat can lead to reduced agility, strength, and flexibility, which can have a negative effect on their performance [45]. In addition, the sexual development and other physical maturation processes that occur during puberty are a result of hormonal changes. The development of sexual characteristics is influenced by the interactions between growth hormones, sex steroid hormones (estrogens in girls and androgens in boys), and the production of insulin-like growth factor I (IGF-I), which lead to changes in body composition and shape, including alterations in the relative proportions of water, muscle, fat, and bone [46,47]. Furthermore, the adolescent period involves the maturation of the hypothalamic-pituitary-gonadal (HPG) axis, which mediates the release of gonadotropins. The HPG axis plays a crucial role in neural reorganization and rapid changes in body composition and size. These neural and somatic developments may indirectly or directly influence physical activity. Hormonal changes during puberty may also contribute to modifying levels of physical activity as part of an effort to maintain energy balance [46,47].

Our results demonstrated that physical performance improved with chronological age. Jumping and sprinting performance showed significant improvements during adolescence. This period of life is associated with the growth of skeletal muscle mass. In boys, the additional development is associated with an increase in circulating testosterone, which induces the selective hypertrophy of type II muscle fibers. Furthermore, the increase in strength during the pubertal period is related to enhancements in the percentage of fast-twitch muscle fibers, muscle fiber diameter, muscle cross-sectional area, and muscle length [48].

This is in accordance with the study performed by Jones and Round [48], showing that an increase in muscle length during growth contributes to power and strength. Moreover, Plotkin et al. [49] found that the variability in muscle size, which is dependent on muscle type, may be related to differences in fiber-type composition and how fiber-type shifting occurs with different types of exercise training.

### 4.2. Maturity and Gender-Specific Anthropometric and Physical Parameters

Our findings demonstrate significant differences among the three maturity categories for anthropometric parameters and physical performance. In fact, late-maturing athletes performed significantly better in various physical performance tests (i.e., the 20-m shuttle run test, CMJ height, SJ height, and *t*-test) compared to the early-maturing athletes in both boys and girls.

Unlike chronological age, maturation is a non-linear process. Sexual and somatic maturation in children varies individually in tempo and timing, which may explain the disparity between maturation and chronological age among youth [50]. Tempo describes how slowly or quickly individuals progress along the path to full sexual maturity. Specifically, adolescents are categorized as slow, average, or fast-maturing, depending on how long it takes them to progress through the sexual maturity categories. In contrast, timing is a measure of the differences among individuals in pubertal development and describes how mature adolescents are relative to their same-sex and same-age peers. Indeed, adolescents are classified as early, on, or late-maturing based on their relative physical maturity [51]. 

Furthermore, it appears that increases in body shape, muscle hypertrophy, and the presence of sexual and growth hormones during puberty can enhance physical performance [48]. Jones and Round [48] demonstrated that boys showed a significant increase in stature, bone density, and muscle mass and a simultaneous reduction in limb fat under the influence of IGF-I and circulating androgens (i.e., testosterone and dehydroepiandrosterone (DHEA)). Consequently, an increase in testosterone levels may contribute to the greater formation and development of fast-twitch muscle fibers, which have a positive impact on explosive muscle actions in handball [48]. 

Additionally, other mechanisms could be involved, such as nerve activation, elastic energy release, intensified excitation-contraction coupling, and improvements in strength transmission to different bone levers [45,48]. Our findings regarding the effects of biological maturity are in accordance with the study conducted by Lesinski et al. [52], which demonstrates that more mature athletes, across various sports, including handball, reveal higher anthropometric parameters and better physical performance than less mature athletes.

These results are in accordance with the findings of Hammami et al. [53], which indicated a greater impact of maturity (pre-peak height velocity vs. post-peak velocity) on agility, sprinting, and jumping performance in 56 male handball players aged 12–14 years. On the other hand, early-maturing players may not be as motivated to excel in physical activity due to their socialization process, unlike late-maturing players, who are often encouraged to participate in sports [54].

In contrast to the previous findings, Matthys et al. [55] reported significant differences in favor of early-maturing Belgian male handball players aged 14 years for anthropometry, sprint, and strength tests when comparing early, on-time, and late-maturity groups.

Aerobic power increases with age during childhood in both sexes, starting from the age of 14 years. Maximal aerobic performance capacity in girls levels off at 14 years, while in boys, it continues to increase until the age of 18 years. In fact, girls’ aerobic power is significantly lower, approximately 15% less. Other growth factors, such as longer levers and greater musculature, continue to develop and influence the effectiveness and mechanical efficiency of aerobic activities. In children, cardiovascular adaptation is similar to that of adults and is equally efficient. More precisely, the muscle structure, glycogen storage mechanics, and values are identical and similar to those of adults [56]. 

Unlike the maturation process, situational factors, such as ethnicity, socioeconomic status, education level, father absence, and social support, along with environmental and nutritional factors, influence the onset of puberty and adolescent development.

### 4.3. Interaction Effects of Age and Maturity

Our analysis revealed significant maturity-by-age interactions for specific physical fitness tests (SJ height, five-jump test, T-agility, and medicine ball throw). Our findings align with a study conducted by Tounsi et al. [57], which demonstrated significant differences in anthropometric variables, leg muscle volume, and soccer-specific tests based on PHV categories. Their statistical analysis showed a significant age-maturity interaction effect on all anthropometric variables in soccer players, although not in handball players. Our findings are in agreement with previous research that has reported results similar to ours but with differences in physical tests. For instance, Selmi et al. [58] established percentiles based on normative data for the repeated sprint ability test for young soccer players in different maturity groups. Additionally, Asadi et al. [59] found that post-PHV soccer players indicated greater gains than pre-PHV in vertical jump tests and sprint performance after training.

### 4.4. Percentile Values According to Maturity Status and Gender

In terms of established maturity and sex-specific percentile reference values for physical tests, there is a lack of studies that provide sex and maturity-specific percentile values for handball players in Tunisia. There are a few studies available for various sports and populations. For example, Tomkinson et al. [60] provided sex and age-specific normative reference values for physical fitness (measuring balance, strength, endurance, power, flexibility, speed-agility, speed, and cardiorespiratory fitness) in European children and adolescents aged 9–17 years. Lesinski et al. [52] established maturity and sex-specific anthropometric and physical fitness percentiles of young elite German athletes across different sports. In Germany, Albrecht et al. [61] provided sex and age-specific normative values for handgrip strength and components of the senior fitness test for older adults. Meanwhile, in China, Ma et al. [62] developed sex and age-specific percentiles for the physical fitness components among Chinese children and adolescents aged 7–18 years. There are similarities between our study and the mentioned studies regarding the establishment of sex and age-specific normative reference values for various physical parameters. However, there are many differences in terms of the participants’ age and the range of physical tests [52,60,61,62].

In Tunisia, Tounsi et al. [42] established normative data for jumping performance in healthy Tunisian adolescents aged 13–19. Furthermore, Aouichaoui et al. [63] provided percentile values specifically for vertical jumping performance in athletic Tunisian children aged 7 to 18 years practicing gymnastics, soccer, handball, volleyball, basketball, swimming, and tennis.

Due to the lack of literature examining maturity-specific anthropometric and physical fitness percentiles for male and female athletes, especially in handball adolescent athletes, our findings are considered preliminary.

Percentiles are valuable not only for establishing the relative position of a value but also for dividing our data into segments, determining central tendencies, and assessing distribution dispersion. Consequently, percentiles serve as an exploratory data analysis tool in descriptive statistics.

There are potential limitations when using percentiles in the context of established maturity and sex-specific percentile reference values for physical tests. In fact, percentiles are based on data from a specific population and may not be applicable to different ethnic, cultural, or geographical groups. Adolescents may progress through puberty at different rates, and percentiles do not always account for these variations. As a result, some individuals may be inappropriately classified based on their chronological age. Additionally, percentiles alone do not provide a complete picture. They do not consider factors like training history, nutrition, injury history, or psychological factors that can influence physical performance. It is important to consider these limitations when using percentile reference values for physical tests and to interpret them cautiously, taking into account the specific context and individual characteristics of the athletes being assessed.

### 4.5. Methodological Considerations

Research in handball is recommended to evaluate players’ performance using various tests that reproduce the specific movement patterns and physiological characteristics inherent to the game [64]. In team handball, players must accelerate, change directions quickly, and engage in physical activities, such as throwing, collisions, and passing. In the present study, performance measurements for vertical jumps were objectively obtained using the OptoJump Next device, which is a validated tool for estimating jump height [65]. The CMJ and SJ tests were selected because they have been identified to be the most reliable and valid tests for evaluating jump height in players [66]. We only selected handball players whose jumping performance was found to be reproducible. The timing for 5 m, 30 m, and 15 × 6 m sprint tests was measured using a photocell gate device, which is the most reliable tool for assessing sprint performance [67]. The aerobic capacity of the handball players was assessed through maximal oxygen uptake (VO_2max_), a widely utilized parameter in sports medicine [67]. The maximal multistage 20-m shuttle run test involves various activity patterns designed to mirror the intermittent activity profile of a handball match. This test was previously validated as a reliable method for estimating the velocity associated with VO_2max_ [68]. Concerning the maturation status of handball players, a maturation index was computed using a valid and non-invasive method to predict years from PHV [69].

## 5. Limitations

The sample size of our study was not a limiting factor when we performed the power analysis because we used the formula for the sample size calculation to determine the necessary sample size. However, a larger sample might have provided a more comprehensive understanding of the relationships between maturity status and physical performance. The larger the sample size, the better the precision and the lower the risk of error. It also ensures a more accurate representation of the total population. Furthermore, the classification of athletes into early, average, and late-maturity groups might have oversimplified the complex concept of biological maturity. Different athletes may experience variations in their growth and development, and a more nuanced categorization might have yielded more accurate results. Data were gathered to establish growth reference values, although ideally, longitudinal data should have been employed. The longitudinal design is preferable as it enables explanatory inferences.

Additionally, there is a possibility of selection bias in the study, as athletes who chose to participate may not accurately represent the entire population of handball players in Tunisia. This bias has the potential to impact the external validity of the findings.

Therefore, future studies should consider expanding the sample size to be larger. Additionally, these studies should incorporate other parameters, such as psychological, social, or coaching-related aspects, which can also influence an athlete’s performance. 

## 6. Conclusions

In summary, our study confirmed that advanced maturation status in handball players is associated with more favorable anthropometric and physical fitness characteristics. Male athletes outperformed females in both anthropometric and performance parameters. This study provides maturity and sex-specific percentile values for physical performance in Tunisian handball players aged 13–19 years.

## 7. Practical Applications

Coaches and trainers can use the percentile values to tailor training programs based on an individual athlete’s maturity status and gender, ensuring that training regimens are optimized to meet the specific needs and capabilities of each player. The assessment of performance in adolescent Tunisian athletes based on empirical data can be instrumental in verifying the effectiveness of specific training programs, identifying highly talented athletes, and protecting adolescent athletes from potential injuries. Additionally, the percentile values serve as a standardized method for evaluating the physical fitness of adolescent handball players. Coaches can objectively assess the performance of individual players or entire teams, pinpointing strengths and areas for improvement. Understanding how physical attributes and performance evolve with maturity status offers valuable information for talent identification and supporting the growth of young athletes through their developmental stages.

## Figures and Tables

**Table 1:** Descriptive statistics of anthropometric variables in Tunisian handball players by age and gender.

Age (Years)		13	14	15	16	17	18	19
Body mass (kg)	Boys	47.48 ± 9.29 *	56.13 ± 7.27 §	62.04 ± 12.53 §	68.98 ± 15.10 *	69.86 ± 10.84 *	76.91 ± 11.81 *	§ 85.03 ± 10.85 *
	Girls	53.92 ± 7.02	55.83 ± 10.57 †	58.81 ± 8.58	59.81 ± 6.88	61.52 ± 6.61	62.70 ± 6.75	65.11 ± 1.29 †
Standing height (cm)	Boys	160.40 ± 9.33	§ 170.52 ± 4.39 *	171.10 ± 7.91 *	§ 177.58 ± 10.48 *	180.31 ± 6.63 *	180.72 ± 5.46 *	183.80 ± 6.47 *
	Girls	165.79 ± 6.71	165.91 ± 6.85	167.17 ± 7.56	167.58 ± 8.09	171.17 ± 2.45†	172.28 ± 1.75	172.69 ± 0.77
Sitting height (cm)	Boys	76.25 ± 5.01 *	§ 83.40 ± 1.89	82.47 ± 5.08	§ 87.87 ± 6.33 *	90.47 ± 4.32 *	90.29 ± 4.01 *	91.75 ± 4.5 *
	Girls	82.87 ± 4.81	85.34 ± 2.80 †	82.72 ± 4.62	85.70 ± 1.25	84.65 ± 3.44	80.64 ± 6.42	83.10 ± 3.11
Leg length (cm)	Boys	84.22 ± 4.94	87.59 ± 3.64 *	88.03 ± 5.04	89.64 ± 7.17	88.69 ± 3.84 *	87.99 ± 1.49	89.76 ± 3.48 *
	Girls	83.99 ± 8.84	80.60 ± 7.50	84.28 ± 8.86 †	84.66 ± 8.82	86.80± 5.56	87.89 ± 1.45	86.54 ± 3.10
Body mass index (kg·m^−2^)	Boys	18.32 ± 2.98	20.21 ± 2.11	20.89 ± 2.81	21.34 ± 2.71	21.14 ± 2.69	23.06 ± 3.00	24.97 ± 2.81 *
	Girls	19.63 ± 2.46	19.95 ± 2.86	21.12 ± 2.79	21.34 ± 2.52	21.32 ± 2.38	21.53 ± 0.50	21.90 ± 0.19
Fat mass (kg)	Boys	6.64 ± 3.60 *	8.72 ± 2.94 *	9.21 ± 4.94 *	7.83 ± 5.36 *	9.61 ± 5.28 *	13.73 ± 4.74 *	18.09 ± 5.73 *
	Girls	11.57 ± 2.56	14.90 ± 7.12	12.68 ± 4.34	12.96 ± 5.08	13.12 ± 4.58	13.28 ± 6.23	14.13 ± 5.08
Fat-free mass (kg)	Boys	41.46 ± 7.99 *	47.90 ± 7.23 *	52.83 ± 8.92 *	61.15 ± 11.59 *	60.26 ± 7.71 *	63.18 ± 8.58 *	§ 67.33 ± 7.06 *
	Girls	47.22 ± 6.67	41.74 ± 10.93 †	46.43 ± 8.55 †	48.42 ± 8.68 †	49.15 ± 5.53 †	51.65 ± 6.3 †	49.43 ± 6.21 †
Body fat percentage (%)	Boys	13.26 ± 5.74 *	15.042 ± 4.55 *	14.15 ± 5.87 *	10.70 ± 5.40 *§	13.23 ± 5.75 *	§ 17.49 ± 4.63 *	20.78 ± 4.19 *
	Girls	19.66 ± 5.21	25.39 ± 12.81 †	21.53 ± 6.56	20.92 ± 6.48	20.82 ± 5.99	19.83 ± 6.66	22.07 ±7.72
Wingspan (cm)	Boys	165.05 ± 12.13 *	168.82 ± 8.17 *	176.01 ± 5.21 *	180.07 ± 9.56 *	181.60 ± 8.15 *	183.81 ± 7.97 *	185.86 ± 6.99 *
	Girls	164.21 ± 7.41	164.71 ± 7.05	167.92 ± 6.60	167.73 ± 8.14	166.08 ± 7.42	167.49 ± 7.27	165.71 ± 9.67
Handspan (cm)	Boys	20.94 ± 1.76	21.62 ± 1.24 *	22.00 ± 1.60	22.21 ± 1.31 *	22.18 ± 1.30 *	22.16 ± 1.43 *	23.23 ± 1.57 *
	Girls	20.42 ± 1.32	20.84 ± 1.25	20.23 ± 1.16	20.48 ± 1.34	20.13 ± 0.80	19.45 ± 1.53	20.24 ± 1.20

Notes: * Significant difference between boys and girls in the same age group (*p* < 0.05). § Boys: age vs. previous age (*p* < 0.05). † Girls: age vs. previous age (*p* < 0.05).

**Table 2:** Descriptive statistics of the physical and physiological performance of Tunisian handball players by age and gender.

Age (Years)		13	14	15	16	17	18	19
VO_2max_ (mL. min^−1^.kg^−1^)	Boys	45.94 ± 1.11 *	47.66 ± 2.54 *	47.92 ± 5.60 *	47.98 ± 4.00 *	48.31 ± 4.81 *	48.76 ± 2.64 *	51.76 ± 3.72 *§
	Girls	41.05 ± 5.19	41.46 ± 1.73	41.52 ± 4.83	41.56 ± 4.39	42.30 ± 3.91	42.48 ± 5.41	43.77 ± 0.00 †
MAV (km.h^−1^)	Boys	11.55 ± 0.06 *	11.56 ± 0.46 *	11.75 ± 1.00 *	12.01 ± 0.71 *	12.37 ± 0.84 *	13.20 ± 0.44 *	13.43 ± 0.43 *§
	Girls	10.26 ± 1.03	10.43 ± 0.10	10.94 ± 0.83 †	11.03 ± 0.75	11.24 ± 0.65	12.49 ± 0.89	12.60 ± 0.01
SJ height (cm)	Boys	21.99 ± 2.54	22.25 ± 2.85 *	§ 25.90 ± 3.85 *	27.00 ± 4.29 *	§ 29.56 ± 4.62 *	31.60 ± 5.21 *	32.24 ± 4.58 *
	Girls	21.14 ± 0.99	22.22 ± 0.20	22.38 ± 2.78	23.77 ± 4.33	24.41 ± 2.68 †	25.13 ± 2.93	26.24 ± 1.20 †
CMJ height (cm)	Boys	23.01 ± 2.05	23.18 ± 3.15	26.04 ± 3.70 *§	28.26 ± 3.54 *	31.07 ± 4.87 *§	32.28 ± 4.16 *	33.91 ± 4.02 *
	Girls	22.46 ± 0.32	22.26 ± 2.03	23.29 ± 2.75	24.90 ± 3.88	25.24 ± 3.11 †	26.17 ± 3.53	27.02 ± 2.71 †
Five Jump test (m)	Boys	9.18 ± 0.96	9.49 ± 0.96 *	10.10 ± 0.97 *	10.41 ± 0.91 *	11.32 ± 1.02 *	11.54 ± 1.11 *	11.80 ± 0.93 *
	Girls	8.30 ± 0.75	8.75 ± 0.33	8.71 ± 0.77	9.22 ± 1.04	8.94 ± 0.78	8.83 ± 0.96	8.88 ± 0.57
Speed 5 m (s)	Boys	1.39 ± 0.11	1.22 ± 0.01 *	1.22 ± 0.15 *§	1.19 ± 0.10 *	1.13 ± 0.15 *	1.12 ± 0.12 *§	1.14 ± 0.12 *
	Girls	1.55 ± 0.21	1.36 ± 0.11 †	1.37 ± 0.17 †	1.38 ± 0.22	1.45 ± 0.21	1.46 ± 0.16	1.39 ± 0.13
Speed 30 m (s)	Boys	3.92 ± 0.14	3.76 ± 0.17	3.67 ± 0.62 *	3.60 ± 0.74 *	3.41 ± 0.28 *§	3.41 ± 0.21 *	3.40 ± 0.63 *
	Girls	4.13 ± 0.31	4.10 ± 0.27	3.98 ± 0.20	3.97 ± 0.18	3.95 ± 0.47	3.93 ± 0.31	3.90 ± 0.21
Agility test (s)	Boys	7.94 ± 0.08 *	7.86 ± 0.74	7.57 ± 0.29	7.49 ± 2.15	7.41 ± 2.11	7.42 ± 2.06 *	5.93 ± 0.27 *
	Girls	9.01 ± 2.26	7.47 ± 0.71	7.43 ± 0.61	8.64 ± 2.56	7.86 ± 1.83	10.51 ± 3.22 †	9.92 ± 2.93
RSA (s)	Boys	7.15 ± 0.44	7.02 ± 0.19	6.51 ± 0.37 *§	6.44 ± 0.36 *	6.25 ± 0.25 *	6.24 ± 0.32 *	6.13 ± 0.26 *
	Girls	7.44 ± 0.46	7.34 ± 0.49	7.30 ± 0.46	7.28 ± 0.40	7.22 ± 0.48	7.18 ± 0.33	7.16 ± 0.41
Medicine ball throw (m)	Boys	2.99 ± 0.36	3.92 ± 0.60 *§	4.03 ± 0.61 *	4.80 ± 0.80 *§	4.84 ± 0.79 *	5.51 ± 0.92 *§	§ 6.41 ± 0.43 *
	Girls	3.17 ± 0.66	3.08 ± 0.50	3.52 ± 0.66	3.32 ± 0.51	3.70 ± 0.84	3.78 ± 0.49	3.32 ± 0.25
Flexibility (cm)	Boys	3.80 ± 2.09	3.18± 4.94 *	3.23 ± 6.60 *	7.03 ± 4.88 *§	7.73 ± 4.11 *	9.04 ± 4.37 *	7.42 ± 3.93 *
	Girls	4.97 ± 3.68	6.12 ± 5.08	6.52 ± 7.30	7.00 ± 4.05	10.38± 5.15	5.43 ± 1.92 †	5.06 ± 1.47

Notes: * Significant difference between boys and girls in the same age group (*p* < 0.05). § Boys: age vs. previous age (*p* < 0.05). † Girls: age vs. previous age (*p* < 0.05). VO_2max_ = maximum oxygen uptake. MAV = maximum aerobic velocity. SJ = squat jump. CMJ = countermovement jump. RSA = repeated sprint ability.

**Table 3:** Anthropometric characteristics (mean ± SD) of Tunisian handball players according to maturity status and gender.

Maturity Status		Early-Maturing	On-Maturing	Late-Maturing
Chronological age (years)	Boys	13.75 ± 0.96	15.31± 1.04	* 17.68 ± 1.03 §
	Girls	14.07 ± 1.49	15.68 ± 1.49	18.11 ± 0.91 ¥
Body mass (kg)	Boys	48.69 ± 8.31 *	63.88 ± 11.98 *	* 77.34 ± 12.33 §
	Girls	56.50 ± 7.85	59.25 ± 8.29	63.25 ± 6.06 ¥
Standing height (cm)	Boys	162.24 ± 8.65 *	173.48 ± 6.27 *	* 182.67 ± 6.69 §
	Girls	167.09 ± 6.41	167.91 ± 7.74	172.14 ± 2.25 ¥
Sitting height (cm)	Boys	76.60 ± 3.91 *	§ 84.81 ± 2.92 *	* 91.95 ± 3.94 §
	Girls	79.10 ± 4.40	83.91 ± 4.15	85.74 ± 2.24 ¥
Leg length (cm)	Boys	85.40 ± 5.95 *	88.54 ± 4.51 *	* 89.11 ± 4.33 §
	Girls	83.16 ± 7.74	85.68 ± 7.81	86.16 ± 4.25 ¥
Body mass index (kg·m^−2^)	Boys	18.42 ± 2.55 *	21.14 ± 2.48	* 22.88 ± 3.17 §
	Girls	20.35 ± 2.71	21.01 ± 2.51	21.51 ± 1.34 ¥
Fat mass (kg)	Boys	15.89 ± 7.57	22.09 ± 11.25 *	* 33.78 ± 13.34 §
	Girls	17.92 ± 9.90	18.95 ± 10.63	21.03 ± 11.78 ¥
Fat-free mass (kg)	Boys	32.87 ± 10.11 *	42.12 ± 12.20	42.21 ± 10.63 §
	Girls	40.94 ± 9.47	40.37 ± 9.57	41.78 ± 9.61 ¥
Body fat percentage (%)	Boys	33.03 ± 14.83 *	33.74± 15.63 *	* 43.54 ± 15.31 §
	Girls	26.12 ± 15.54	29.69 ± 16.73	30.55 ± 18.99
Wingspan (cm)	Boys	166.69 ± 10.25 *	175.72 ± 8.31 *	* 184.28 ± 7.62 §
	Girls	162.41 ± 6.13	167.00 ± 7.74	168.12 ± 7.49 ¥
Handspan (cm)	Boys	21.09 ± 1.64 *	22.04 ± 1.34 *	* 22.54 ± 1.48 §
	Girls	20.09 ± 1.50	20.25 ± 1.22	20.29 ± 1.30

Notes: § significant difference between early-maturing, on-maturing, and late-maturing status in boys (*p* < 0.05); * significant difference between boys and girls in the same category; ¥ significant difference between early-maturing, on-maturing, and late-maturing status in girls (*p* < 0.05). Early-maturating (−3 years > −1 years from PHV), on-maturing (−1 to +1 years from PHV), late-maturing (>1 to +3 years from PHV).

**Table 4:** Physical performance (mean ± SD) of Tunisian handball players according to maturity status and gender.

Age (Years)		Early-Maturing	On-Maturing	Late-Maturing
VO_2max_ (mL. min^−1^·kg^−1^)	Boys	48.16 ± 2.79 *	48.52 ± 4.33 *	* 48.57 ± 4.70
	Girls	41.05 ± 5.19	41.31 ± 4.06	41.34 ± 3.99
MAV (km·h^−1^)	Boys	11.58 ± 0.46 *	11.93 ± 8.36 *	* 12.57 ± 0.88 §
	Girls	10.26 ± 1.03	10.98 ± 0.77	11.37 ± 0.64 ¥
SJ height (cm)	Boys	23.00 ± 3.23	25.93 ± 4.73 *	* 30.42 ± 4.95 §
	Girls	22.36 ± 3.04	22.50 ± 3.24	22.87 ± 2.49
CMJ height (cm)	Boys	23.88 ± 2.80	26.66 ± 4.68 *	* 31.48 ± 4.49 §
	Girls	23.42 ± 3.02	22.96 ± 3.32	23.48 ± 3.07
Five Jump test (m)	Boys	9.38 ± 0.97 *	10.21 ± 1.09 *	* 11.43 ± 1.05 §
	Girls	8.48 ± 0.98	8.81 ± 0.82	9.03 ± 0.82 ¥
Speed 5 m (s)	Boys	1.30 ± 0.11 *	1.22 ± 0.16 *	* 1.16 ± 0.12 §
	Girls	1.45 ± 0.22	1.41 ± 0.19	1.38 ± 0.13 ¥
Speed 30 m (s)	Boys	3.86 ± 0.30 *	3.94 ± 0.56	* 3.52 ± 0.51 §
	Girls	4.12 ± 0.31	3.99 ± 0.25	3.99 ± 0.29 ¥
Agility test (s)	Boys	7.84 ± 0.76 *	7.88 ± 1.55	* 7.07± 1.87 §
	Girls	8.91 ± 2.28	8.24 ± 2.23	9.28 ± 2.61 ¥
RSA (s)	Boys	6.88 ± 0.47 *	6.65 ± 0.44 *	* 6.22 ± 0.28 §
	Girls	7.46 ± 0.46	7.29 ± 0.44	7.26 ± 0.35 ¥
Medicine ball throw (m)	Boys	3.33 ± 0.64	4.32 ± 0.76 *	* 5.51 ± 0.95 §
	Girls	3.18 ± 0.59	3.44 ± 0.60	3.56 ± 0.56 ¥
Flexibility (cm)	Boys	3.17 ± 4.51	4.28± 5.82 *	* 7.96 ± 4.20 §
	Girls	4.79 ± 5.06	7.18 ± 5.44	6.00 ± 3.12 ¥

Notes: § significant difference between early-maturing, on-maturing, and late-maturing status in boys (*p* < 0.05); * significant difference between boys and girls in the same category; ¥ significant difference between early-maturing, on-maturing, and late-maturing status in girls (*p* < 0.05). Early-maturating (−3 years > −1 years from PHV), on-maturing (−1 to +1 years from PHV), late-maturing (>1 to +3 years from PHV). VO_2max_ = maximum oxygen uptake; MAV = maximum aerobic velocity; SJ = squat jump; CMJ= countermovement jump; RSA = repeated sprint ability.

**Table 5:** Results of the MANOVA analyses examining the differences in anthropometric variables according to age category and/or maturity status per test cluster.

	MANOVA Age * × Maturity		MANOVA Age		MANOVA Maturity	
	[F (*p*)]	η^2^	[F (*p*)]	η^2^	[F (*p*)]	η^2^
Body mass (kg)	916 (0.493)	0.012	2074 (0.055)	0.024	19,891 (*p* < 0.001) *	0.072
Standing height (cm)	3743 (0.001) *	0.049	1343 (0.236)	0.015	23,982 (*p* < 0.001) *	0.086
Sitting height (cm)	4627 (*p* < 0.001) *	0.059	18,645 (*p* < 0.001) *	0.179	201,265 (*p* < 0.001) *	0.440
Leg length (cm)	1822 (0.081)	0.024	2444 (0.024) *	0.028	1490 (0.226)	0.006
Body mass index (kg·m^−2^)	1019 (0.417)	0.014	3068 (0.006) *	0.035	7161 (0.001) *	0.027
Fat mass (kg)	1593 (0.135)	0.021	3633(0.002) *	0.041	7914 (*p* < 0.001) *	0.030
Fat-free mass (kg)	2435 (0.018) *	0.032	1808 (0.096)	0.021	6249 (0.002) *	0.024
Body fat percentage (%)	2198 (0.033) *	0.029	3454 (0.002) *	0.039	6083 (0.002) *	0.023
Wingspan (cm)	1753 (0.095)	0.023	3568 (0.002) *	0.040	43,136 (*p* < 0.001) *	0.144
Handspan (cm)	2175 (0.035) *	0.029	5234 (*p* < 0.001) *	0.058	26,325 (*p* < 0.001) *	0.093

Notes: multivariate F statistics. * indicates significant results (*p* < 0.05). In the case of non-significant multivariate test results, univariate test results were not shown.

**Table 6:** Results of the MANOVA analyses examining the differences in physical performance according to age category and/or maturity status per test cluster.

	MANOVA Age * × Maturity		MANOVA Age		MANOVA Maturity	
	[F (*p*)]	η^2^	[F (*p*)]	η^2^	[F (*p*)]	η^2^
VO_2max_ (mL.min^−1^·kg^−1^)	1586 (0.137)	0.021	2155 (0.046)	0.025	6245 (0.002) *	0.024
MAV (km·h^−1^)	1867 (0.073)	0.025	4287 (*p* < 0.001) *	0.048	7099 (0.001) *	0.027
SJ height (cm)	2552 (0.014) *	0.034	1963 (0.069)	0.022	21,642 (*p* < 0.001) *	0.078
CMJ height (cm)	1967 (0.058)	0.026	2033 (0.060)	0.023	22,064 (*p* < 0.001) *	0.079
Five jump test (m)	3430 (0.001) *	0.045	3513 (0.002) *	0.039	33,575 (*p* < 0.001) *	0.116
Speed 5 m (s)	1612 (0.129)	0.022	8204 (*p* < 0.001) *	0.088	9543 (*p* < 0.001) *	0.036
Speed 30 m (s)	1112 (0.354)	0.015	3149 (0.005) *	0.036	15,656 (*p* < 0.001) *	0.058
Agility test (s)	6727 (*p* < 0.001) *	0.084	13,852 (*p* < 0.001) *	0.139	21,810 (*p* < 0.001) *	0.078
RSA (s)	0.833 (0.560)	0.011	3097 (0.005) *	0.035	22,890 (*p* < 0.001) *	0.082
Medicine ball throw (m)	2619 (0.012) *	0.035	0643 (0.696)	0.007	33,960 (*p* < 0.001) *	0.117
Flexibility (cm)	1935 (0.062)	0.026	5593 (*p* < 0.001) *	0.061	0.481 (0.619)	0.002

Notes: multivariate F statistics. * indicates significant results (*p* < 0.05). In the case of non-significant multivariate test results, univariate test results were not shown. VO_2max_ = maximum oxygen uptake; MAV = maximum aerobic velocity; SJ = squat jump; CMJ = countermovement jump; RSA = repeated sprint ability.

**Table 7:** Specific percentile values for physical performance according to maturity status and gender.

		Boys							Girls						
	Maturity Status	5%	10%	25%	50%	75%	90%	95%	5%	10%	25%	50%	75%	90%	95%
VO_2max_ (mL. min^−1^.kg^−1^)	Early	43.25	45.00	46.70	49.70	49.70	50.84	52.20	33.46	35.64	38.65	41.80	45.30	48.70	54.50
	On	40.50	43.20	45.30	48.20	50.90	54.10	55.34	34.22	36.54	39.40	42.80	45.30	46.70	47.50
	Late	41.60	42.30	45.30	48.90	51.90	55.10	55.87	31.10	35.80	41.60	42.30	42.40	45.30	46.80
MAV (km.h^−1^)	Early	10.78	11.08	11.50	11.60	11.60	11.70	12.60	8.55	9.40	10.05	10.50	11.45	12.10	13.00
	On	10.50	10.96	11.60	11.60	12.50	13.14	13.36	9.76	10.00	10.40	11.00	11.60	12.00	12.10
	Late	11.16	11.50	11.90	12.60	13.40	13.68	13.90	9.80	10.30	11.50	11.60	11.60	11.88	12.40
SJ height (cm)	Early	18.89	19.32	20.80	22.70	25.25	27.06	29.40	18.25	19.52	21.09	21.31	23.92	25.26	29.42
	On	18.28	20.28	22.90	25.30	28.80	32.68	35.20	17.80	18.66	19.40	21.31	24.00	25.86	26.91
	Late	23.10	23.87	26.80	29.90	33.70	37.44	39.46	18.18	18.83	18.83	21.10	23.92	23.92	24.37
CMJ height (cm)	Early	20.00	20.28	21.80	23.40	25.45	28.34	28.82	19.26	19.90	22.15	22.83	25.53	26.47	30.18
	On	19.38	20.68	23.70	25.55	29.90	32.66	34.76	17.48	18.78	20.14	22.83	25.53	26.74	28.27
	Late	24.17	25.55	28.05	31.40	34.50	37.20	38.97	17.76	18.38	20.14	22.20	25.55	25.55	26.50
Five-jump test (m)	Early	7.69	7.98	8.75	9.30	10.05	10.73	11.11	7.23	7.26	7.75	8.50	8.90	10.51	10.68
	On	8.49	9.00	9.21	10.30	11.00	11.54	12.11	7.41	7.81	8.28	8.70	9.40	9.82	10.68
	Late	9.65	10.27	10.68	11.40	12.13	12.71	13.47	7.79	8.01	8.40	9.10	9.40	10.68	10.68
Speed 5 m (s)	Early	1.06	1.13	1.22	1.30	1.39	1.45	1.46	1.00	1.03	1.19	1.41	1.55	1.70	1.74
	On	0.94	0.99	1.13	1.25	1.38	1.40	1.41	1.06	1.12	1.28	1.43	1.58	1.65	1.72
	Late	0.99	1.06	1.06	1.16	1.27	1.31	1.37	1.22	1.28	1.37	1.43	1.56	1.65	1.68
Speed 30 m (s)	Early	3.44	3.55	3.71	3.82	3.98	4.10	4.51	3.68	3.74	3.90	4.12	4.34	4.52	4.79
	On	3.22	3.32	3.53	3.86	4.08	4.94	5.09	3.58	3.70	3.84	4.02	4.16	4.33	4.46
	Late	3.04	3.07	3.21	3.39	3.68	4.52	4.67	3.65	3.70	3.79	3.97	4.12	4.37	4.50
Agility test (s)	Early	6.67	6.89	7.35	7.72	8.31	8.31	9.05	6.67	6.85	7.33	7.80	11.32	12.82	13.48
	On	5.88	6.03	6.80	7.55	8.31	10.41	11.49	6.36	6.51	7.00	7.51	8.19	13.04	13.66
	Late	5.53	5.73	5.87	6.26	7.31	10.52	11.15	6.74	6.90	7.17	7.72	12.51	13.96	15.01
RSA (s)	Early	6.03	6.19	6.54	6.96	7.22	7.45	7.70	6.58	7.03	7.19	7.32	7.88	8.02	8.52
	On	5.98	6.04	6.26	6.64	7.09	7.22	7.24	6.54	6.70	6.96	7.28	7.60	7.90	8.08
	Late	5.82	5.87	5.98	6.19	6.41	6.66	6.74	6.56	6.66	7.06	7.31	7.46	7.68	7.82
Medicine ball throw (m)	Early	2.50	2.56	2.84	3.16	3.85	4.34	4.34	2.30	2.50	2.63	3.30	3.79	3.94	4.12
	On	3.11	3.29	3.80	4.34	4.70	5.46	5.87	2.50	2.64	3.10	3.45	3.89	4.35	4.41
	Late	4.10	4.27	4.72	5.57	6.12	6.91	7.11	2.72	2.96	3.22	3.45	4.10	4.45	4.62
Flexibility (cm)	Early	−7.72	−1.56	1.40	3.80	5.10	8.42	9.28	−6.42	0.62	1.95	5.57	8.15	12.14	13.30
	On	−8.76	−2.88	0.80	4.50	7.60	11.64	12.60	0.02	1.26	3.20	5.70	10.70	14.80	16.62
	Late	0.66	2.32	5.35	8.10	10.50	13.78	15.77	1.00	1.72	4.40	5.57	7.00	11.96	13.10

Notes: VO_2max_ = maximum oxygen uptake; MAV= maximum aerobic velocity; SJ = squat jump; CMJ = countermovement jump; RSA = repeated sprint ability.

## Data Availability

Data are available from the corresponding author upon reasonable request.
